# Active interfacial dynamic transport of fluid in a network of fibrous connective tissues throughout the whole body

**DOI:** 10.1111/cpr.12760

**Published:** 2020-01-19

**Authors:** Hongyi Li, Yajun Yin, Chongqing Yang, Min Chen, Fang Wang, Chao Ma, Hua Li, Yiya Kong, Fusui Ji, Jun Hu

**Affiliations:** ^1^ Beijing Hospital National Center of Gerontology Beijing China; ^2^ Department of Engineering Mechanics Tsinghua University Beijing China; ^3^ Department of Human Anatomy, Histology and Embryology Neuroscience Center Institute of Basic Medical Sciences Chinese Academy of Medical Sciences Beijing China; ^4^ School of Basic Medicine Peking Union Medical College Beijing China; ^5^ Institute of Computing Technology Chinese Academy of Sciences Beijing China; ^6^ Key Laboratory of Interfacial Physics and Technology Shanghai Institute of Applied Physics Chinese Academy of Sciences Shanghai China; ^7^ Shanghai Synchrotron Radiation Facility Shanghai Advanced Research Institute Chinese Academy of Sciences Shanghai China

**Keywords:** extracellular matrix, interstitial fluid, interstitium, vascular circulations

## Abstract

Fluid in interstitial spaces accounts for ~20% of an adult body weight and flows diffusively for a short range. Does it circulate around the body like vascular circulations? This bold conjecture has been debated for decades. As a conventional physiological concept, interstitial space is a micron‐sized space between cells and vasculature. Fluid in interstitial spaces is thought to be entrapped within interstitial matrix. However, our serial data have further defined a second space in interstitium that is a nanosized interfacial transport zone on a solid surface. Within this fine space, fluid along a solid fibre can be transported under a driving power and identically, interstitial fluid transport can be visualized by tracking the oriented fibres. Since 2006, our data from volunteers and cadavers have revealed a long‐distance extravascular pathway for interstitial fluid flow, comprising at least four types of anatomic distributions. The framework of each extravascular pathway contains the longitudinally assembled and oriented fibres, working as a fibrorail for fluid flow. Interestingly, our data showed that the movement of fluid in a fibrous pathway is in response to a dynamic driving source and named as dynamotaxis. By analysis of previous studies and our experimental results, a hypothesis of interstitial fluid circulatory system is proposed.

## 1 INTRODUCTION

This paper will review some studies on interstitial fluid (ISF) flow and summarize our experimental discoveries in active interfacial dynamic transport of ISF. This active ISF transport is of distinguished characteristics below: (a) It is long‐ranged instead of short‐ranged. (b) It is systemic instead of regional. (c) It is ordered instead of random. (d) It is structuralized, multilayered, multi‐levelled, multiscaled, fibre‐oriented and interfacial‐constrained. (e) It is power‐controlled, that is heart‐beating‐driven or periodic‐power‐source‐driven. (f) It is a faster transport process rather than a slower diffusion process, that is the measured peak flow speed is approximately 2 cm/s. (g) It is systemically cycled, that is the exchanges with vascular circulations and the dynamic cycled patterns are universally exist. Finally, a hypothesis of interstitial fluid circulatory system is proposed.

## 2 THE TRADITIONAL VIEWPOINTS ABOUT ISF FLOW

The total body fluids in adult can be divided into three compartments: (a) the fixed intracellular fluid compartment that accounts for about 32% of body weight, (b) the systemically circulatory fluid compartment (blood plasma, lymphatic fluid and cerebrospinal fluid) for about 8% of body weight, and (c) the local‐transport interstitial fluid compartment for about 20% of body weight. It is noted that the word “local‐transport” or “diffusive transport” is used when ISF is mentioned in literatures.

In physiology, ISF is thought to be entrapped within the interstitial/extracellular matrix and transports diffusively for a very short‐ranged.^1^ Generally, the composition of interstitial matrix comprises two phases: the fluid/gel phase, consisting of the combination of proteoglycan filaments and ISF entrapped within them, so called tissue gel, and the solid phase, consisting of a three‐dimensional cross‐linked network of fibres embedded within tissue gel. The interstitial matrix in biology is usually regarded as porous materials, and the ISF transport through the fluid/gel phase is believed to obey the Darcy's law. When the concentration gradient or pressure gradient is present, ISF randomly and diffusively transports water and solutes in a short‐ranged space among cells, blood capillaries and initial lymphatic vessels to modulate cellular environment in tissues. Although almost all ISF is normally entrapped within tissue gel, a part of ISF can still be free of the proteoglycan molecules and flow randomly and locally in the short‐ranged regions. The following phenomenon has been recorded in the textbook of physiology: when a dye is injected into the circulating blood, it often can be seen that ISF flows freely through interstitium, usually coursing along the surfaces of collagen fibres or the surfaces of cells.^2^

Roughly speaking, a few key words about the characteristics of traditional ISF diagram may be summarized: entrapped, localized, short‐ranged, disordered, permeated, single‐level structured, single‐size scaled, and concentration‐gradient driven or pressure‐gradient driven, Darcy's law controlled. However, such localized diagram of ISF flow has been debated for several decades.

## 3 REPRESENTATIVE STUDIES RELATED TO ISF TRANSPORT IN THE HISTORY

Could the ISF transport systematically throughout the whole body? As far as we know, there are three representative studies on the ISF transport beyond vascular circulations in history.

The earliest record of a long‐distance ISF transport is “the fluid in perivascular spaces” described in 1851 by Rudolph Virchow, which is an extravascular channel for ISF flow between the outer and inner lamina of brain vessels or around fenestrated capillaries. This diagram is further studied recently in researches on the clearance pathways of the brain. A perivascular drainage of ISF along the basement membrane of smooth muscle cells of intracerebral and cerebral arteries and capillaries has been observed by identifying the deposits of amyloids.^3^ A paravascular drainage pathway of ISF along arterial and venous walls has been assessed by injection of fluorescein and labelled Aβ in brain.^4^ By rapid distribution of tracer protein throughout the brain from the subarachnoid space, fluid circulation through central nervous system via paravascular pathways has been proposed.^5^ Basically, the perivascular and paravascular pathways are regarded as a channel around blood vessels. However, their topographical connections with the heat, the directions of flow, the detailed histological structures of the perivascular and paravascular spaces, their exact relationship with the arteries, veins and capillaries are elusive.

The second is the interstitial tissue channel. Around the 1970s, by injection of ferrocyanide ions into blood vessels from which they leak into the interstitial tissues, it was found that the precipitate deposits of ferric ions were in the connective tissues near vessel wall or in the skeletal muscle tissues under the electron microscope, light microscope and in the mesentery of rabbit and cat by dark‐field transilluminations.^6^, ^7^, ^8^ To understand the transport of the fluid and the ionic tracer through the extravascular interstices in fibrous connective tissues, a water‐rich region in extravascular connective tissues was proposed and named as “prelymphatic tissue channel” by G. Hauck or “interstitial tissue channel” by Casley‐Smith. The interstitial tissue channel forms a converging drainage system from the arterial side of capillaries towards their venous sides and the initial lymphatics nearby. In some tissues like brain and retina where is lacking in lymphatics, the interstitial tissue channels can be very long and perform a function of the prelymphatic pathways from the deep portions of brain tissues to the lymphatic vessels in the neck. However, the tissue channels and their surrounds cannot be stained quantitatively and cannot be directly visualized by electron microscope either. Until now, the space structures of tissue channel and the detailed transport processes of ISF flow through the gel‐like interstitial matrix have not been fully understood.

The third is the explorations on the Meridian and Collateral Channel network described in traditional medical literatures. Around the 1980s, instead of intravenous injection, an isotopic tracer was hypodermically injected into an acupuncture point in hands or feet of humans.^9^ The centripetal transport processes of the isotopic tracer from the hypodermic injection points were clearly visualized under the single‐photon emission computerized tomography (SPECT). The isotope trajectories suggested an extravascular pathway originated from an acupuncture point in the extremities. Another similar phenomenon of the long‐distance ISF flow from an acupuncture point has been studied by the injection of Trypan blue or Alcian blue and attributed to “Bonghan ducts” or “Primo‐vascular system,” through the vascular conduits of which, ISF can flow.^10^ However, the microscopic structures of primo‐vascular‐conduit are unclear yet.

The above experiments are heuristic: (a) By comparison with the complicated measurements of the interstitial pressure gradients, it is more convenient to identify interstitial fluid flow by an imaging tracer. (b) To visualize the extravascular ISF transport pathways, the imaging tracer shall be injected into a certain region of interstitial tissues outside the vascular vessels. (c) The strategy to identify a tubular structure for fluid flow may be ineligible. Accordingly, a few essential questions need to be answered: (a) How to clarify the anatomical and histological structures of the long‐distance ISF transport pathways in humans? (b) What are the mesoscopic and microscopic structures conducting the ISF flow? (c) What is the transport mechanism of the long‐distance ISF flow? (d) What is the role of the beating heart in ISF flow?

## 4 OUR EXPERIMENTAL DISCOVERIES ABOUT THE SYSTEMIC ISF TRANSPORT

Since 2006, our research group has made a series of progresses exploring the ISF transport. Different from the isotopic tracers, we use several techniques with higher spatial resolution including contrast‐enhanced MRI and have visualized a long‐distance extravascular transport pathway from a hypodermic point in the extremity endings of volunteers, usually an acupuncture point.^11^ After the hypodermic injection of Gd‐DTPA into the acupuncture points in the hand and foot of human subjects, two types of the long‐distance extravascular pathways were enhanced by the paramagnetic tracer in the extremities: the pathways with unsmoothed continuous trajectories and the pathways with smooth continuous trajectories.^11^, ^12^ If the injection point on the hand or foot is in the vicinity of a venous vessel but not an acupuncture point, only the smooth pathways could be displayed.^11^, ^12^ Further analysis of the imaging data revealed that the smooth pathways seemed to have enhanced partial walls of the venous vessel other than the intravascular lumen.^12^ The unsmoothed pathways from acupuncture points were in the subcutaneous tissues and have a characteristic of “puncture resistance from acupuncture needle.”^11^, ^12^ Through lymphangiography by the hypodermic injection of iodized oil into an acupuncture point, we found that neither the smooth nor the unsmoothed pathways were in accordance with the visualized lymphatic vessels.^12^

### 4.1 The anatomical and histological structures of an extravascular pathway from an acupuncture point in humans

Another valuable progress from our research group is the discovery of the anatomical and histological structures of the long‐distance extravascular pathways from an acupuncture point, especially in physiological conditions. One subject was recruited who had severe gangrene foot due to arteriosclerosis obliterans and would receive the amputation of his right lower leg. BEFORE the amputation, he took the hypodermic injection of the fluorescent tracer into an acupuncture point in ankle. Around 90 minutes after the amputation, tissue samples from the lower leg were histological analysed. It was clearly visualized by the fluorescein that four types of the extravascular pathways from the ankle to the amputated end of leg were consistently formed by fibrous connective tissues^13^: (a) a cutaneous pathway in the dermis and hypodermis tissues, (b) a perivenous pathway along the venous adventitia (including the adventitia), (c) a periarterial pathway along the arterial adventitia (including the adventitia), (d) a fibrous‐endoneurium‐perineurium‐epineurium pathway in the nerves. These results clearly demonstrate that the ISF transport pathways are of multiformity and have at least four types of anatomical distributions. Conventional concept of tissue channel is insufficient to sum up such multiformity. We conjecture that the smooth pathways observed in volunteers by MRI were probably the perivascular pathways and the unsmoothed pathways were the cutaneous pathways.

In addition to physiological conditions, ISF transport pathways in non‐physiological conditions were also investigated. Three amputated lower legs received the injection of the fluorescent tracer into the same acupuncture point were investigated AFTER their amputation. In these cases, periodic “mechanical compressions” on the amputated end of the legs were covered by a sphygmomanometer cuff with a systolic pressure of 50‐60 mm Hg and a compression‐relaxation frequency of 18‐20 times/min. After around 90 minutes of manipulation, the same four types of the fluorescently stained fibrous pathways were observed.^13^

The ISF transport pathways discovered above in both physiological and non‐physiological conditions may lead to the following judgements: (a) The long‐distance ISF transports include at least four types of anatomical structures in living human body or the cadavers. (b) The histological structures of each ISF transport pathway are consistently formed by fibrous connective tissues which suggest that ISF flow universally exists in fibrous matrices throughout the whole body. (c) The transport mechanisms of a long‐distance ISF transport were not fully understood yet but might be periodically power‐sources‐driven or multi‐driving‐centres‐driven. The to‐and‐fro movements of the dynamic source are of vital importance in ISF transport, like the pulsation of the heart and the vasomotions of vascular vessels.

We have disclosed some optical imaging features of the long‐distance ISF transport as well. Using two‐photon confocal laser microscopy (TPCLSM), all the extravascular pathways were found to contain thousands of the fluorescently stained and micron‐sized fibres being distributed longitudinally along the long axis of the transport pathways.^13^ The following findings may be concluded: (a) The stained fibres are the results of the fluorescent ISF flow through the extravascular pathways and there must be a fluid film on a fibre. (b) The space between the fibres is surrounded by the gel substances and there must be a constrained space for fluid film flow along a fibre. (c) The longitudinal fibres (or fibril‐bundles) play a role as a guiderail for fluid flow in the oriented interstitial connective tissues. (d) Because the distributions of the fibres are long‐ranged and oriented, the ISF transport is long‐ranged and ordered accordingly. In short, as a fibrous guiderail (fibrorail), the fibres are of essential importance in ISF transport through a highly structuralized interstitial matrix.

To further confirm the universal existence of the above discoveries in the whole body, the human cadavers were used to testify the extravascular pathways by the simulated heart pulsation using a mechanical chest compressor.^14^ Upon the hypodermic injection of the fluorescent tracer into an acupuncture point in the first knuckle of thumb, a fluorescently stained extravascular pathway from the right thumb to right atrium near chest wall was visualized after 2.5‐hours of repeated chest compressions. The cutaneous pathways from the thumb were found in dermic, hypodermic and fascial tissues of the hand and the lower forearm, but not found in the skin above the level of fossa cubitalis. The intrinsic millimetre‐sized structures of the cutaneous pathways and the controlled skin tissues outside the pathways were observed, respectively, by micro‐CT. Within these cutaneous pathways, the millimetre‐sized interlobular septum of hypodermic adipose tissues was oriented towards the transport direction. By contrast, the hypodermic interlobular septum outside the cutaneous pathways is irregular. The perivascular pathways from the thumb were observed along the veins of the arm, axillary sheath, superior vena cava and in the superficial tissues on the right atrium. Histological and micro‐CT (a higher resolution) data showed that these long‐distance pathways were neither blood nor lymphatic vessels but the fibrous connective tissues, in which the micron‐sized fibres were longitudinally assembled from the thumb to the superficial tissues on the right atrium and appendage. These anatomical data verified that the structural framework of the fibrous pathways is composed of the multilayered, longitudinally assembled and cross‐linked micron‐sized fibres, which provide a fibrorail network to guide ISF flow directionally. By TPCLSM, it was clearly observed that these connective tissues comprise the abundant fluorescently stained fibres oriented along the long axis of the transport pathway, which were the results of the fluorescent fluid flow along the fibrorails.

### 4.2 An interfacial transport zone within interstitial matrices and an interfacial dynamic transport pathway at a multiscale

In interstitial connective tissue, neither the fibre nor the gel themselves can flow.^1^ Based on the above experimental evidences at macroscopic and mesoscopic scales, we can construct the ISF transport space as follows: the fluorescently stained micron‐sized fibres in fibrous matrix are an *in situ* imaging evidence of a fluid film paved on the fibre by the optical microscope. There are no other visible features in this transport region except for the visualized fibres and gel under the submicron resolution. From the viewpoint of interface science, an interfacial space would be formed physically between two phases of matters. In interstitial matrix, the only possible transport space for ISF flow must be an *interfacial transport zone* (ITZ) between the fibre (a solid phase) and the gel/liquid substance (a liquid phase).^15^ The ITZ is not an interface of a few molecular layers but contains a thin, probably a nanometre‐thickness space paved on a solid surface.^14^ The space shape of an ITZ mainly depends on the solid surfaces of a fibre, a cell, a bundle of fibres, or a group of cells, and may be variform in a biological system. In a transverse section view, an ITZ has two types of the interfaces: an interface on a solid surface of fibres or cells, and an interface in contact with the gel phase of matrix. The pore sizes or compartment sizes of the ITZs might be variable in physiological or pathophysiological conditions, which may be a nano‐scale space paved on a fibre or even a micron‐scale space paved on a layer of cells. Both of an ITZ and its contacting gel play as a basic unit of kinetic and dynamic transport processes for fluid flow.^14^ Indeed, the detailed microscopic structures of the ITZ and the interactions between the ITZ and the gel matrix need further studies.

In macroscopic scale like the anatomical structures, the microscopic ITZs can be topologically connected along the intrinsic fibres and comprise a macroscopic transport pathway of long‐ranged characteristics throughout the whole body. When ISF comes into the topologically connected ITZs, ISF may continuously flow via these oriented interstitial connective tissues by a driving power and present two types: ISF flow along the embedded, oriented and ordered fibrorails and in the meanwhile diffuse/exchange between the ITZs and the surrounding tissue gel along the long‐distance extravascular pathways.^13^ This ISF transport pattern is named “*interfacial dynamic transport* (IDT).” According to the anatomical positions, the IDT pathways can be classified into different types, such as a cutaneous IDT pathway, a neural IDT pathway, a facial IDT pathway, an adventitial IDT pathway, an extracellular IDT pathway, a tumorous IDT pathway, a neoplastic IDT pathway, an interosseous IDT pathway, a glandular IDT pathway, etc^14^ Just like the long journey of vascular circulations since the 17th century, the understandings of an active IDT network need in‐depth studies to both the anatomical/microscopic structures and functions of the systemic ISF flow and is critical to comprehend fluid circulations in life system.

### 4.3 Imaging of the adventitial ISF flow *in vivo*

In rabbits, a continuous fluid flow in both the adventitial pathway and its surrounding fibrous connective tissues as well as their topographical connections with the heart has been observed *in vivo*.^16^ Visualized by the fluorescent tracer that was injected into the interstitial spaces around the arterial and venous vessels in the ankle, the fluorescent ISF from the ankle was driven along the adventitia and its surrounding fibrous connective tissues of vasculature, into the atrioventricular, anterior and posterior interventricular grooves of the heart, forming pericardial fluid in the rabbits. At the same time, the peripheral fluorescent ISF was found in the walls of segmental small intestines and right pulmonary veins as well, which indicated that there was probably more than one centre of IDT driving powers in physiological conditions. The measured speed of venous adventitial ISF flow was around 0.2‐2 cm/s in the rabbit.^16^ By Doppler technique, the measured speed of femoral arterial blood flow in the rabbit was around 5‐50 cm/s and the femoral venous blood flow was around 6‐7 cm/s.^17^ Usually, the diffusion speed from a capillary to a cell is around 1 mm/s.^18^ As far as we know, the visualized ISF flow is faster than diffusion and seems to fill in the gap of velocity spectrum for body fluid from a lowest diffusion to a faster blood flow.

A bulk‐like emerging flow in the adventitial IDT pathway has been dynamically recorded by fluorescence microscope in *in vivo* rabbits in our laboratory. Firstly, when an incision was made in a segmental venous adventitia, continuous ISF flow can be observed in the incision area and exhibit a continuous bulk‐like emerging flow of ISF in the ITZs along the fibres of the adventitial IDT pathway. Secondly, when the fluorescein in the IDT pathways began to fade away, a few fluorescent plaques formed by dozens to over one hundred micrometres were found to flush away, that probably represented a bigger ITZ on layers of smooth muscle cells between the tunica adventitia and tunica media of vascular vessels (Figure 2E1,E2). These imaging data are a distinguished phenomenon of fluid flow via multi‐sized and multi‐form ITZs in living animals, which may comprehend our understandings on the transport functions of each layer of vascular vessels.^3^

### 4.4 The adventitial ISF flow exchanges with blood circulation via capillaries

Is the long‐distance continuous ISF flow cycled? According to classical physiology, the ISF is generated by the filtration of plasma from arterial capillaries and retaken by venous capillaries and lymphatics. Our data in the rabbit demonstrated that the long‐distance adventitial IDT pathways played a role to deliver the peripheral ISF into the pericardial cavity to form pericardial fluid and spread/exchange ISF into the surrounding tissues along vascular tree.^16^ At the same time, the fluorescent tracer from the IDT pathways was found to stain the venous valves inside the inferior vena cava, which indicated that the fluorescent tracer entered the blood circulation eventually. By tracing the whereabouts of the tracers in the adventitial IDT pathways under the fluorescence microscopy, it was easily found that the fluorescent ISF in an IDT pathway has been retaken by the capillary net and converged into a venous vessel nearby. The results coincide with the understanding of ISF exchange with capillaries that was described in 1896 by Ernest Starling. Thus, the continuous ISF flow can exchange constantly with blood circulation via the capillaries among the long‐distance IDT system, including the capillaries in coronary circulation, and circulate around the body.

## 5 VASCULATURE'S KINETIC LAWS FOR FLUID FLOW

Our findings suggest Vasculature's Kinetic Laws for fluid flow: (Ⅰ) Fluid transport via vascular vessels comprises two types: an intravascular blood flow and an adventitial ISF transport. (Ⅱ) For venous vessels, the direction of adventitial ISF transport is the same as that of blood flow. (Ⅲ) For arterial vessels, the direction of adventitial ISF transport is opposite to that of blood flow.

In addition, we define that an adventitial ISF/IDT transport includes three parts (Figure 2B,C): (1) A paravascular/perivascular pathway in the surrounding tissues along tunica adventitia of the vasculature. (2) An ISF/IDT pathway through tunica adventitia of the vasculature. (3) An ISF/IDT pathway between the tunica adventitia and the surfaces of layers of smooth cells of tunica media.

For the arteriole, venule and capillaries in the diverse visceral organs including the brain, the ISF flow directions of each part of an adventitial ISF/IDT pathway need to be identified. According to our data, the to‐and‐fro movements of the beating heart play a special role as a driving centre or transferring centre in regulating fluid flow in an adventitial ISF/IDT pathway. Within these driving centres, like the beating heart and the respirometric lung, ISF flow in each part of an adventitial ISF/IDT pathway needs to be clarified, respectively. The lymphatic vessel, structured like blood vessels, comprises endothelial cells, a thin layer of smooth muscle and fibrous adventitia that binds the lymphatic vessels to the surrounding tissues. We predict that there is an adventitial ISF/IDT transport in the lymphatic adventitia as well.

## 6 A HYPOTHESIS OF INTERSTITIAL FLUID CIRCULATORY SYSTEM

**Figure 1:**
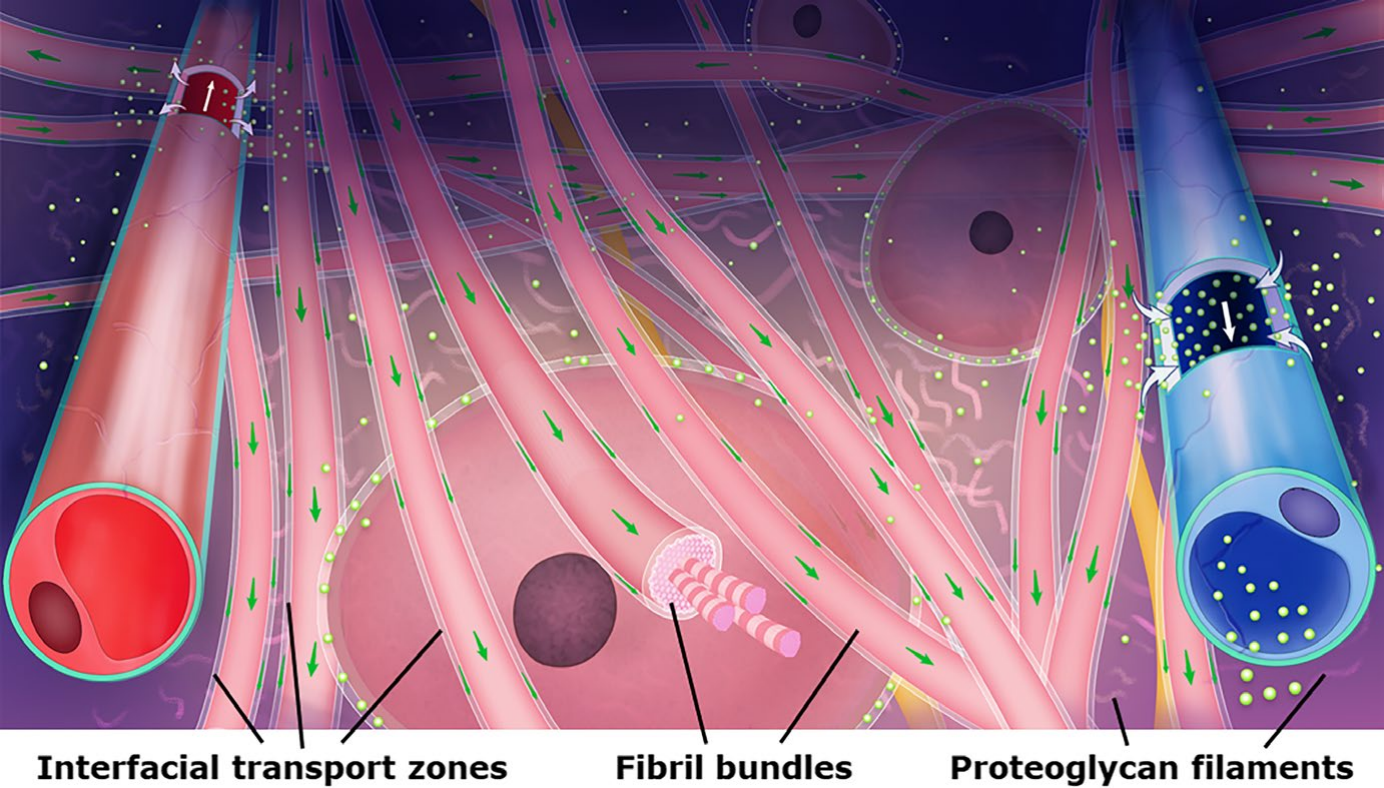
**Illustration of interstitial fluid diffusion and interfacial fluid transport along fibrorails in interstitium**. Derived from an arterial side of the capillary, interstitial fluid (green dots) diffuses into the gel‐like substance among fibres and is reabsorbed by the venous side of the capillary, which is a conventional concept of interstitial fluid exchange in microcirculation. In the meanwhile, fluid enters into an interfacial transport zone (ITZ) on a fibre and forms interfacial fluid. Interfacial fluid (green arrows) can be transported along the fibrorails of extracellular matrices under an active dynamic driving power. The collagenous fibres are hydrophilic and participate in the formation of the ITZs. Whether there is an ITZ on the hydrophobic elastic fibres needs to be further clarified. By contrast to the bulky and irregular interstitial space between cells and vasculature, the fine ITZ provides a constrained, ordered and oriented space for fluid flow

In lights of the revealed second space in interstitium and structural framework of fibrous connective tissues throughout the whole body, the diagram of fluid flow through fibrous matrices are renewed (Figure 1): (a) The interstitial matrix should not be viewed as a random porous media, but as a three‐dimensional network of highly ordered, multilayered and cross‐linked fibres embedded with a gel substance. (b) At microscopic scale, fluid flow through fibrous matrix should not only be viewed as a diffusive transport process governed by Darcy's law, but also an interfacial transport process among the *interfacial matrix* driven by an active dynamic mechanism. (c) Fluid flow through fibrous matrices is of multiscale characteristic. An ITZ on a fibrorail is microscopic but the topologically connected ITZs on the multilayered, highly ordered and cross‐linked fibrorails are macroscopic and long ranged. In an extravascular pathway with a diameter of millimetres and a length of centimetres or even over one metre, fluids in the ITZs would be a bulk‐like continuous flow under a driving power. (d) At macroscopic scale or a topographical anatomy, fluid flow through fibrous matrices constitutes an extremely complex network of a long‐distance IDT pathway from the extremity endings to visceral organs or among organs or tissues or cells throughout the whole body. (e) The transport processes of the IDT pathway network are “active and dynamic.” Without the powers, fluid in matrices would diffuse locally. The movement of fluid in an IDT pathway is in response to a dynamic driving source or centre, which is named as dynamotaxis. Usually, the dynamotactic direction of fluid flow in an IDT pathway is towards a driving source or centre. (f) It is noted that the driving sources and centres are various other than one unique driving centre. One of the driving sources in an IDT system has been proposed to be the dynamic to and fro “gel pump.”^14^ The detailed kinetic and dynamic mechanisms to modulate fluid in an IDT system are waiting‐to‐be‐explored in an alive biological system and especially humans. (g) Beyond cardiovascular circulation, a network of the IDT pathways may provide a different solution to systemically transport fluids for a very long distance with a fast and vast velocity around the body of animals and humans, carrying oxygen, nutrients, metabolic products, heat, biological substances, extracellular signalling molecules, bio‐signals, acupuncture signals, ions and the nanoparticles with sizes of dozens nanometres, etc (h) The IDT system might not only be a transport system but also an interfacial dynamic communicating system (IDC), communicating and regulating signal exchanges among each part of IDT system by means of need‐to‐be‐understood mechanisms. In our opinion, the IDC system might be modulated systemically also via the continuous daily movements of regular heart beatings, irregular respiration and skin evaporation, etc (i) Fascinatingly, the reasons of why the heart drives ISF into the external tissues of the heart and the whereabouts of ISF being transferred by the heart remain unclear. (j) The exact physiological and pathophysiological functions of an active IDT/IDC system need to be comprehensively identified. (k) The electrical potential differences caused by ion flow of interfacial fluid transport via ITZs might generate action potential beyond the membrane of a cell, the electrochemical interactions and physiological roles of which are extremely intriguing. (l) Based on our previous findings of a nerve fascicle in lower leg that was stained by fluorescent fluid from a far distance,^13^ we hypothesize there is a continuous ISF or endoneurial fluid flow through an endoneurium, perineurium and epineurium around axons by means of an active IDT towards a driving centre. The conventional diagram of nervous system may be remodified as a complex network that coordinates the electrochemical signals by neurons and glial cells as well as fluids transport by a neural IDT/IDC pathway, communicating the brain and spinal cord and various parts of the body. The relationships between a neural IDT/IDC and axon functions need further studies.

**Figure 2:**
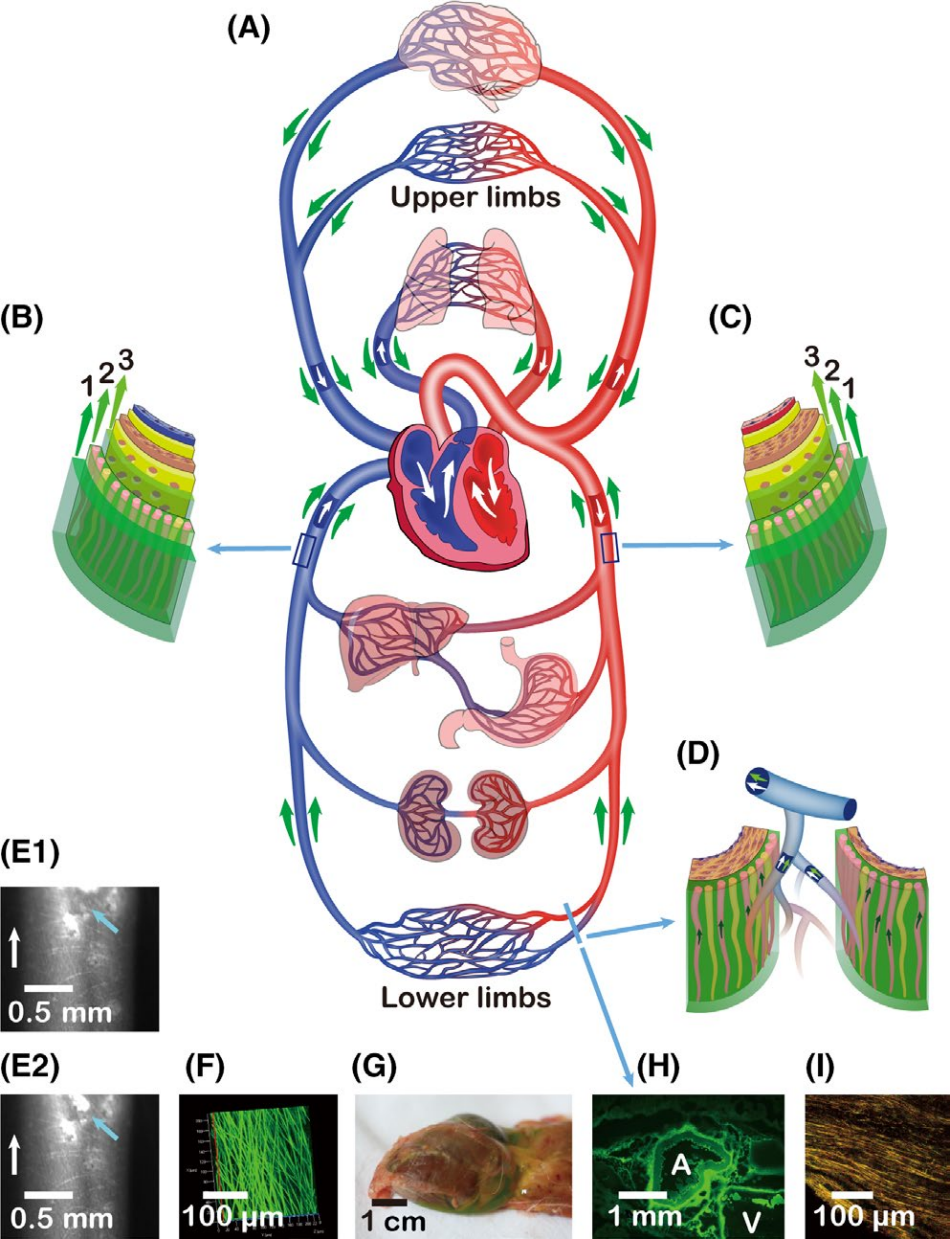
A diagram of an adventitial ISF/IDT system that illustrates a part of the hypothesized interstitial fluid circulatory system. **A** shows: (Ⅰ) Fluid transport via vascular vessels comprises two types: an intravascular blood flow (white arrows) and an adventitial ISF transport (green arrows). (Ⅱ) For venous vessels, the direction of adventitial ISF transport is the same as that of blood flow. (Ⅲ) For arterial vessels, the direction of adventitial ISF transport is opposite to that of blood flow. **B** (a segment of venous wall) and **C** (a segment of arterial wall) show that there are three types of an adventitial ISF transport: 1, fluid flow through paravascular/perivascular spaces along tunica adventitia; 2, fluid flow through tunica adventitia; 3, fluid flow between the tunica adventitia and tunica media. **D** shows that the fluid in an adventitial IDT pathway is taken by capillaries nearby and converges into vascular circulation eventually. The reabsorption of ISF by capillaries occurs in any capillary bed of all parts of the body, including coronary vasculature. **E1** shows a fluorescent plaque of 0.2mm (pointed by a blue arrow) in an adventitial IDT pathway of a venous vessel under a fluorescence stereomicroscope. **E2** shows the fluorescent plaque (blue arrow) flushes away few seconds after **E1**. The findings of **E1** and **E2** strongly suggest there is a bigger interfacial transport zone between the adventitia and media of the vessel, the 3rd type of an adventitial IDT pathway. Under confocal microscope, **F** shows the adventitia of the inferior vena cava was stained by the fluorescent fluid from ankle dermis of a rabbit. **G** shows the excessively accumulated pericardial fluid of the heart that was taken an injection of 4‐6mL fluorescent fluid into ankle dermis of a rabbit.^16^ Under fluorescence microscopy, **H** shows the adventitia and its surrounding tissues of a vein (**V**) and an artery (**A**) in the leg were stained by the fluorescent fluid from ankle dermis of a rabbit.^16^ Under confocal microscope, **I** shows that the adventitia of an arterial vessel in the amputated leg was stained by the fluorescent fluid from ankle dermis of a human^13^

And above all, the understandings on interstitial fluid are renewed as well: (1) The interstices of interstitium or interstitial space may refer to a steric space between the cells. In this bulky space, the majority components of interstitial space are an extracellular matrix that is mainly composed of types of collagenous and elastic fibres, and glycosaminoglycans which form a gel‐like substance. In other words, the matrix in interstitial space contains a structural three‐dimensional scaffolding embedded in the gel‐like interstitium. Firstly, we have further defined a fine space within this structuralized interstitium as an *interfacial transport zone* paved on a solid surface, like a fibrorail or a layer of cells. Fluid in an ITZ can transport along the solid surfaces under an active dynamic driving power, the transport pattern of which is named as an *interfacial dynamic transport*. Secondly, the intrinsic structural framework of interstitium is NOT irregular but ordered. By contrast to the bulky and irregular interstitial space between cells and vasculature, the fine ITZ provides a constrained, ordered and oriented space for fluid flow. Molecules and solutes in interstitial spaces would execute not only “random walks” by diffusion but also “oriented transport” along ordered fibrorails. (2) Based on the fundamental definitions of ITZ and IDT/IDC, systemic fluid flow in fibrous matrices may be reconstructed. (3) The conventional definition of interstitial fluid may refer to fluid in the interstitial compartment that accounts for ~ 20% of body weight as well as fluid in the interstitium between the cells and vasculature. Parts of interstitial fluid, which is fluid in an ITZ of interstitial matrices, may be redefined as interfacial fluid. (4) Like the above analysis, interfacial fluid can transport or flow systemically around the whole body at multiscales, multi‐levels and multi‐layers, such as an adventitial IDT network, a neural IDT network, a cutaneous IDT network, an facial IDT network, etc (5) The movements of ISF is from and back to blood circulation. Parts of ISF enter the ITZs and are transported by an IDT pathway. Fluid in an IDT pathway can quickly and easily interchange with ISF and eventually exchange with vascular circulations by capillary beds or other unidentified ways, that might be the adventitia of an arterioluminal vessel or thebesian veins. (6) Here, we make a hypothesis: (a) Besides diffusive transport in short range, interstitial and interfacial fluid circulate through all parts of the body, which is named as an interstitial/interfacial fluid circulatory system (IFCS) and abbreviated as interstitial fluid circulation. (b) The interstitial and interfacial fluid in extracellular/interstitial matrices, the blood and lymph in vascular circulations comprise three principal compartments of the circulatory system in animals and humans, which together constitute a multiplex circulatory system. (7) Like blood and lymph flow under the actions of the heart, fluid in an adventitial ISF/IDT system (Figure 2), which is a part of IFCS, is driven by the to and fro movements of the heart as well. (8) Unlike vascular circulations, the topographical structures and diagram of fluid flow in the other IDT systems remains unclear, such as the neural IDT system and its driving centres, the cutaneous IDT system and its driving centres, the fascial IDT system and its driving centres, etc (9) The comprehensive understandings of IFCS and its exact relationships with vascular systems, nervous system and all the other anatomical or functional biological systems need to be further clarified.

Particularly, further anatomic studies are indispensable. We propose an international cooperation project for Human Interstitial Fluid Connectome Atlas (HICA) to identify the detailed anatomical and microscopic structures of the entire IFCS and IDT system by a method of dynamic topographical anatomy and a guidance of traditional medicine. The medical applications might benefit from any progress in the understandings on the IFCS and HICA, such as medical imaging technique, a novel pathway for drug delivery, therapeutic instruments, etc. Future studies will need an interdisciplinary cooperation to reveal the regulating mechanisms of IFCS and its relationships with therapeutics. Our pilot studies may provoke a new field of fascinating and fruitful researches in life science.

## CONFLICT OF INTEREST

The authors declare no competing financial interests and have no conflicts to disclose.

## AUTHOR CONTRIBUTIONS

HyL conceived the original ideas, conceptions and designed the experiments and analysis. HyL, QcY, CM and YyK performed the gross human anatomic and animal experiments and the histological analysis. HyL, MC, FW, FsJ and HL analysed the MRI data. HyL, YjY and JH analysed the interfacial transport pattern. HyL wrote the manuscript. All authors contributed to scientific discussions of the manuscript.

## Data Availability

The data that support the findings of this study are derived from the public domain resources. Some detailed data that support the findings of this study are also available from the corresponding author.
